# Supplementary material for: First steps into the cloud: Using Amazon data storage and computing with Python notebooks
Source: PLoS One. 2023 Feb 9;18(2):e0278316. doi: 10.1371/journal.pone.0278316 (PMC9910747; doi:10.1371/journal.pone.0278316)
Supplement: S1 File — (PDF) [file pone.0278316.s001.pdf]

NOV 05, 2022

SHARE

WORKS FOR ME 1

Steps for setup of AWS organization, S3 data storage, and EC2 computing for using Python notebooks

DOI
dx.doi.org/10.17504/protocols.io.rm7vz3z4xgx1/v1

Daniel J. Pollak[1], Gautam Chawla[1], Andrey Andreev[1], David A. Prober[1]

[1]Division of Biology and Biological Engineering, Tianqiao and Chrissy Chen Institute for Neuroscience, California Institute of Technology, Pasadena, California, United States of America

Andrey Andreev: Corresponding author

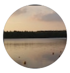

Dan P

COMMENTS 0

ABSTRACT

With the oncoming age of big data, biologists are encountering more use cases for cloud-based computing to streamline data processing and storage. Unfortunately, cloud platforms are difficult to learn, and there are few resources geared towards biologists for demystifying them. We have developed a guide for experimental biologists to set up cloud processing on Amazon Web Services to cheaply outsource data processing and storage. Here we provide a guide on setting up a computing environment in the cloud and showcase examples of using Python and Julia programming languages. We present example calcium imaging data in the zebrafish brain and corresponding analysis using suite2p software. Tools for management of users and budgets are discussed in the protocol. Following this guide should help researchers even with limited programming experience to get started or move existing coding infrastructure into the cloud environment.

DOI

dx.doi.org/10.17504/protocols.io.rm7vz3z4xgx1/v1

PROTOCOL CITATION

Daniel J. Pollak, Gautam Chawla, Andrey Andreev, David A. Prober 2022. Steps for setup of AWS organization, S3 data storage, and EC2 computing for using Python notebooks. **protocols.io**
https://dx.doi.org/10.17504/protocols.io.rm7vz3z4xgx1/v1

FUNDERS ACKNOWLEDGEMENT

NIH
Grant ID: R35 NS122172

NIH
Grant ID: T32 NS105595

KEYWORDS

Python, AWS, cloud, computing

LICENSE

This is an open access protocol distributed under the terms of the Creative Commons Attribution License, which permits unrestricted use, distribution, and reproduction in any medium, provided the original author and source are credited

CREATED

Oct 14, 2021

LAST MODIFIED

Nov 05, 2022

Citation: Daniel J. Pollak, Gautam Chawla, Andrey Andreev, David A. Prober Steps for setup of AWS organization, S3 data storage, and EC2 computing for using Python notebooks https://dx.doi.org/10.17504/protocols.io.rm7vz3z4xgx1/v1

This is an open access protocol distributed under the terms of the **Creative Commons Attribution License** (https://creativecommons.org/licenses/by/4.0/), which permits unrestricted use, distribution, and reproduction in any medium,

PROTOCOL INTEGER ID

54102

SAFETY WARNINGS

Using "cloud" computing can lead to budget overruns due to pay-after-use nature of AWS and other providers. Consult with your home IT department on how to best manage costs and deployment of software.

Setting organization and budget management

1 Setting up a Root account

1.1 Create "root" account for your organization, using Business account type

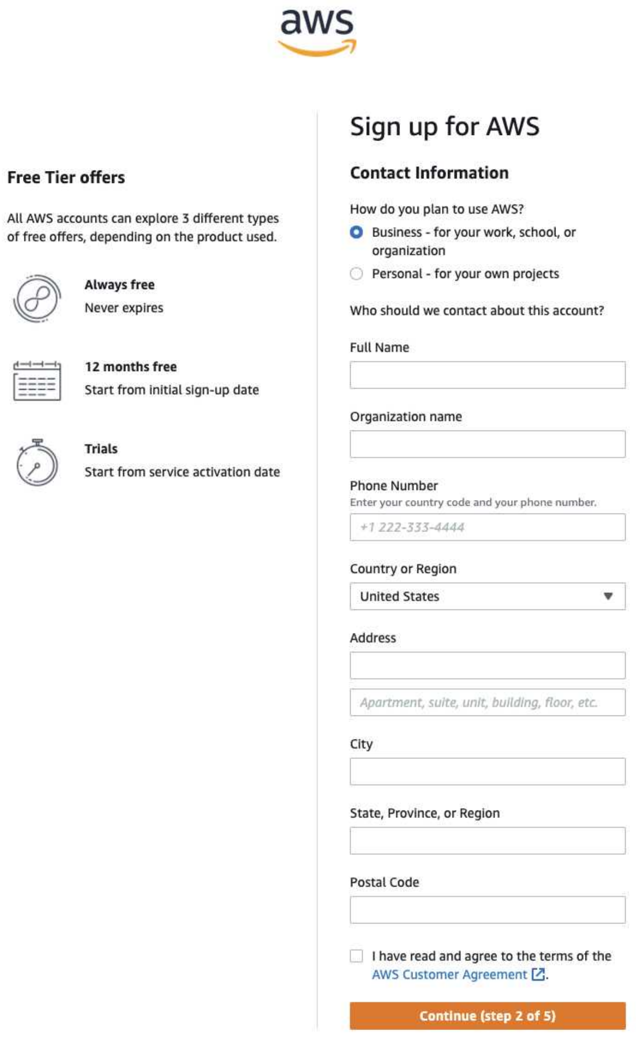

Screenshot from https://portal.aws.amazon.com/billing/signup?type=enterprise#/account

1.2 If you are using GMail account to manage other services in your lab, for example labname@gmail.com, you can use labname+aws@gmail.com to register account with AWS (so-called "gmail + trick")

1.3 You will have to enter credit card information to register root account. Consult your department if you want to avoid using personal credit card

Citation: Daniel J. Pollak, Gautam Chawla, Andrey Andreev, David A. Prober Steps for setup of AWS organization, S3 data storage, and EC2 computing for using Python notebooks https://dx.doi.org/10.17504/protocols.io.rm7vz3z4xgx1/v1

This is an open access protocol distributed under the terms of the **Creative Commons Attribution License** (https://creativecommons.org/licenses/by/4.0/), which permits unrestricted use, distribution, and reproduction in any medium,

2 Research credits can be applied to the account of the organization. Contact entity that issued the credits (most likely your IT department)

3 There are two options to allow users be part of the organization.
External account can be added to organization, but it might be better to [create accounts within organization interface:](#)

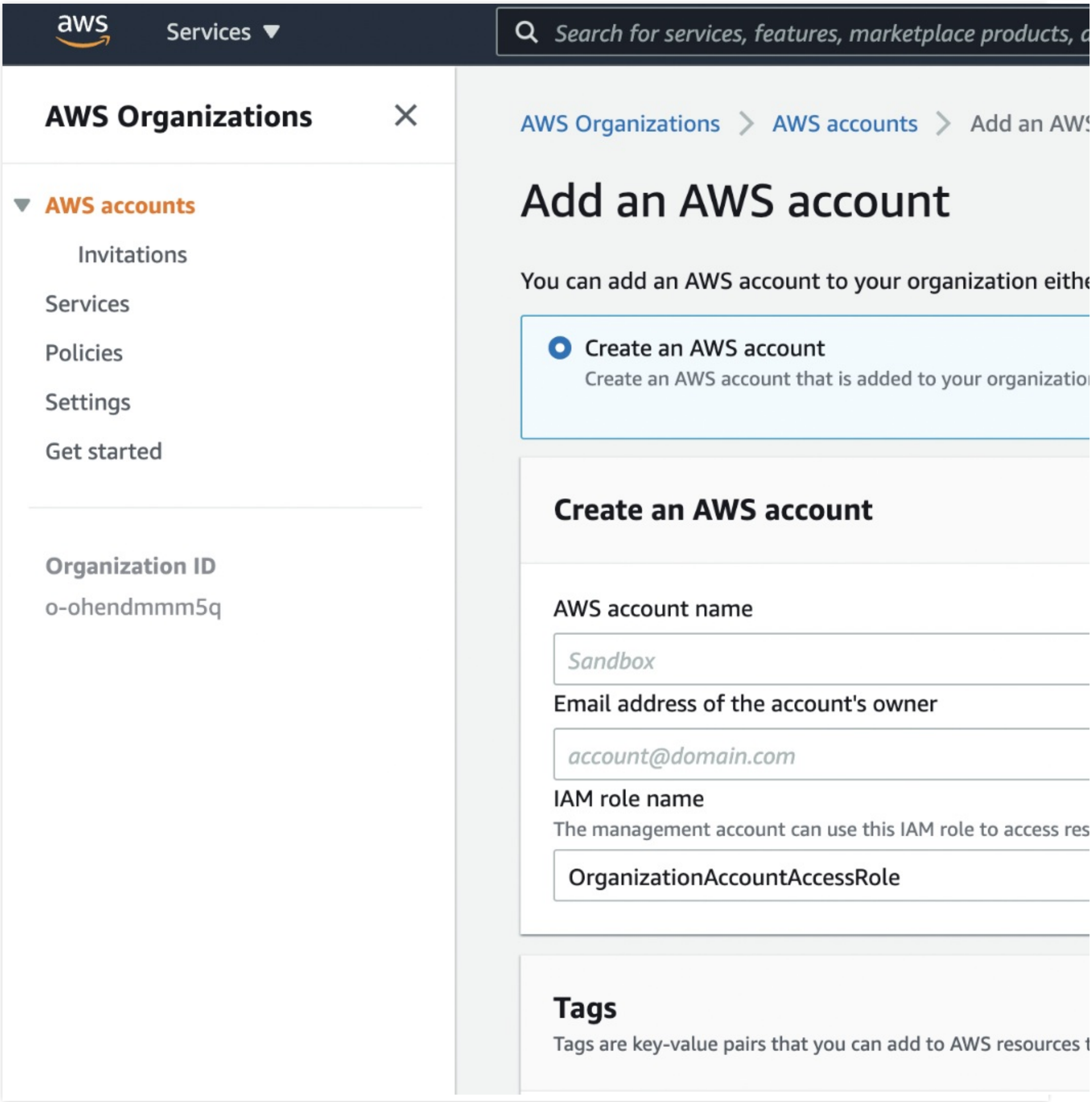

4 After an account has been created, lab member should receive email and use the “reset password” function to set up new password.

5 Each member of the organization can have an individual budget set up. Monthly cost budget is a good starting point to manage costs on a per-account basis.

Citation: Daniel J. Pollak, Gautam Chawla, Andrey Andreev, David A. Prober Steps for setup of AWS organization, S3 data storage, and EC2 computing for using Python notebooks https://dx.doi.org/10.17504/protocols.io.rm7vz3z4xgx1/v1

This is an open access protocol distributed under the terms of the **Creative Commons Attribution License** (https://creativecommons.org/licenses/by/4.0/), which permits unrestricted use, distribution, and reproduction in any medium,

Because sometimes cost can skyrocket accidentally due to misconfigured software (by the users) budget alerts and limits will provide safeguard against such overruns

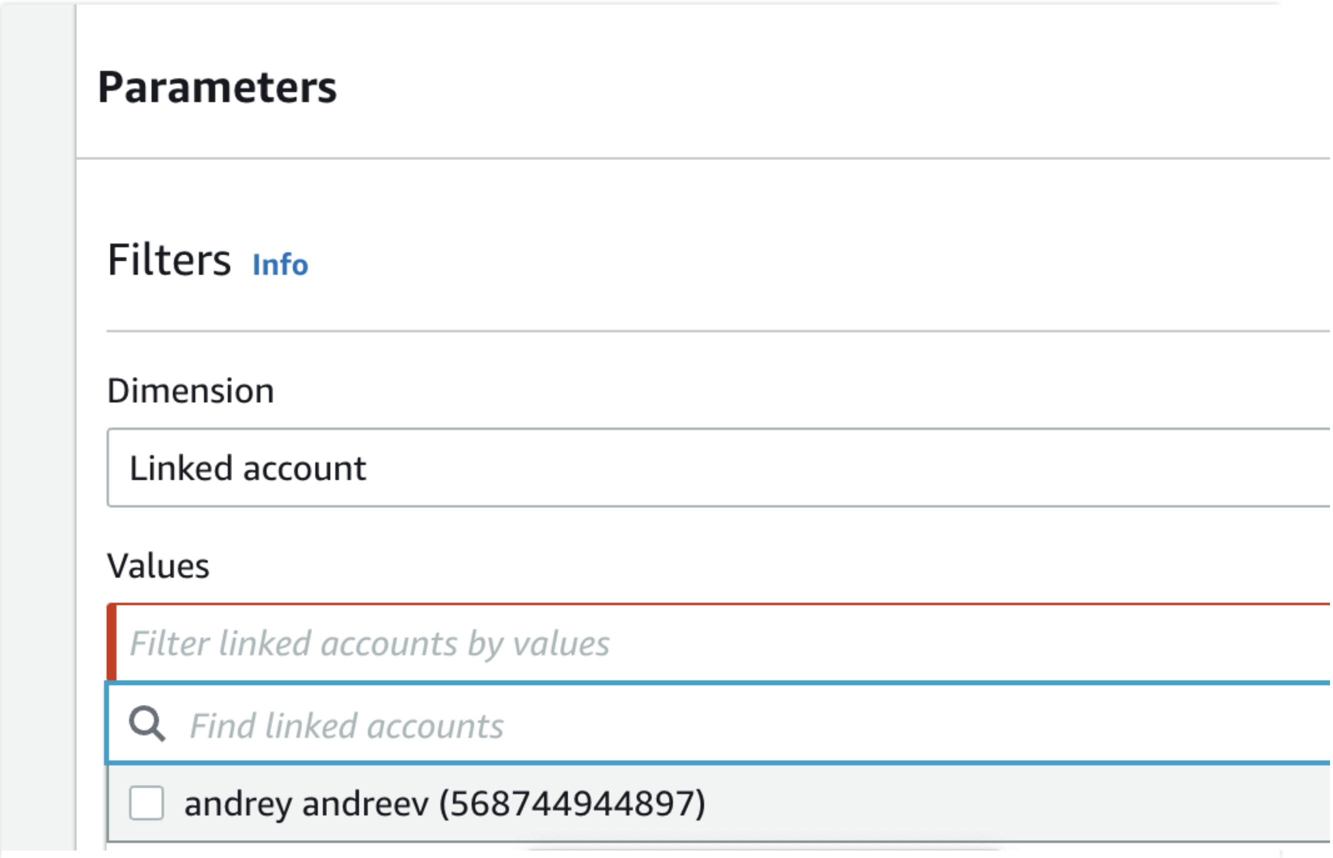

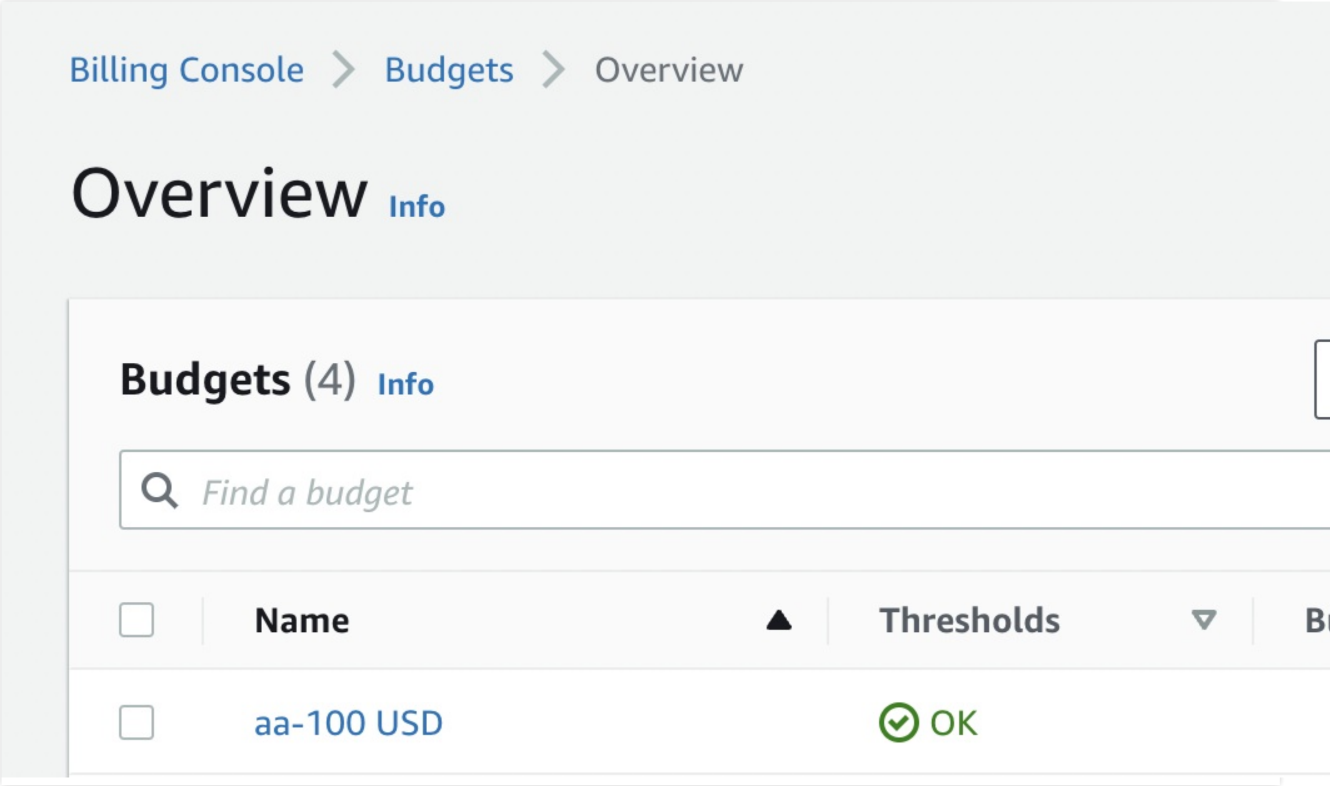

6 It is simple to check the spending by individual account using Cost Explorer in the organization Billing Dashboard.
You can select account (Linked Account). It is important to also pick correct Charge Type (most commonly you can use Credit)

Citation: Daniel J. Pollak, Gautam Chawla, Andrey Andreev, David A. Prober Steps for setup of AWS organization, S3 data storage, and EC2 computing for using Python notebooks https://dx.doi.org/10.17504/protocols.io.rm7vz3z4xgx1/v1

This is an open access protocol distributed under the terms of the **Creative Commons Attribution License** (https://creativecommons.org/licenses/by/4.0/), which permits unrestricted use, distribution, and reproduction in any medium,

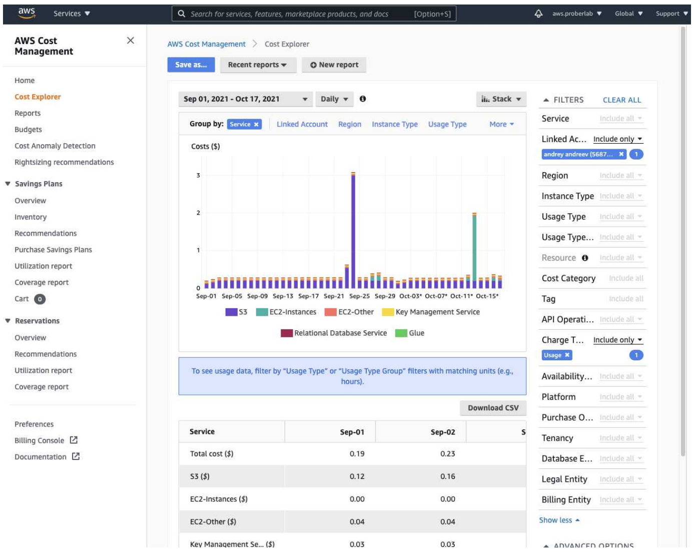

EC2: Starting up a computing instance

7 We consulted two tutorials to set up computing instances:

1. By Justin Bois, the AWS setup and usage lesson in his Caltech BE/Bi 103b class "Statistical Inference in the Biological Sciences" https://bebi103b.github.io/lessons/08/aws_setup.html
2. By Chris Albon: Run Project Jupyter Notebooks On Amazon EC2

8 Find a new instance image (operating system) from the **AWS marketplace**, either Amazon Linux 2, or Ubuntu 18.04 or 20.04.
We recommend using Amazon Linux 2

9 On the Launch page, in the dropdown Choose Action menu, choose Launch through EC2

Citation: Daniel J. Pollak, Gautam Chawla, Andrey Andreev, David A. Prober Steps for setup of AWS organization, S3 data storage, and EC2 computing for using Python notebooks https://dx.doi.org/10.17504/protocols.io.rm7vz3z4xgx1/v1

This is an open access protocol distributed under the terms of the **Creative Commons Attribution License** (https://creativecommons.org/licenses/by/4.0/), which permits unrestricted use, distribution, and reproduction in any medium,

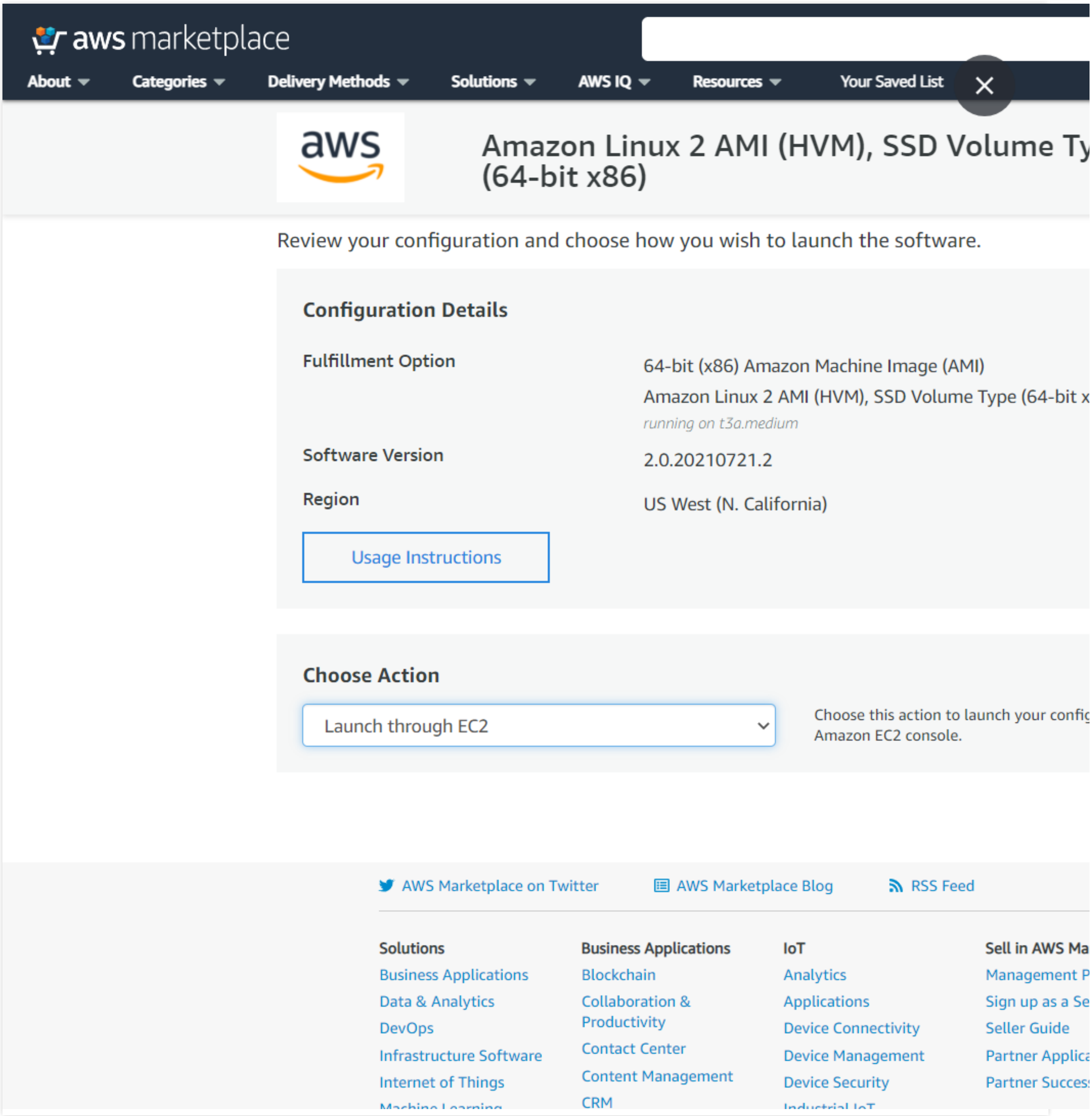

10 This will open a Launch wizard. In the Launch Instance wizard, you will be brought through a 5 part launch sequence.

10.1 **Chose AMI**

Amazon Machine Image (AMI) is the blueprint of the operating system (OS). It can be Linux, FreeBSD, or even Windows. We recommend Amazon Linux 2 for this guide. Later on in this guide we discuss creating your own, personalized, private Amazon Machine Images.

10.2 **Choose Instance Type.**

This will configure your virtual machine "hardware" such as memory size and CPU.
Some relevant parameters here are memory size (RAM), number of cores, and internet speed. For most applications, RAM should be at least 32 GB, the number of cores should be at least 8, and the internet should be very fast, especially because we will be streaming tens or even hundreds of GB of data from S3 to EC2.

Citation: Daniel J. Pollak, Gautam Chawla, Andrey Andreev, David A. Prober Steps for setup of AWS organization, S3 data storage, and EC2 computing for using Python notebooks https://dx.doi.org/10.17504/protocols.io.rm7vz3z4xgx1/v1

This is an open access protocol distributed under the terms of the **Creative Commons Attribution License** (https://creativecommons.org/licenses/by/4.0/), which permits unrestricted use, distribution, and reproduction in any medium,

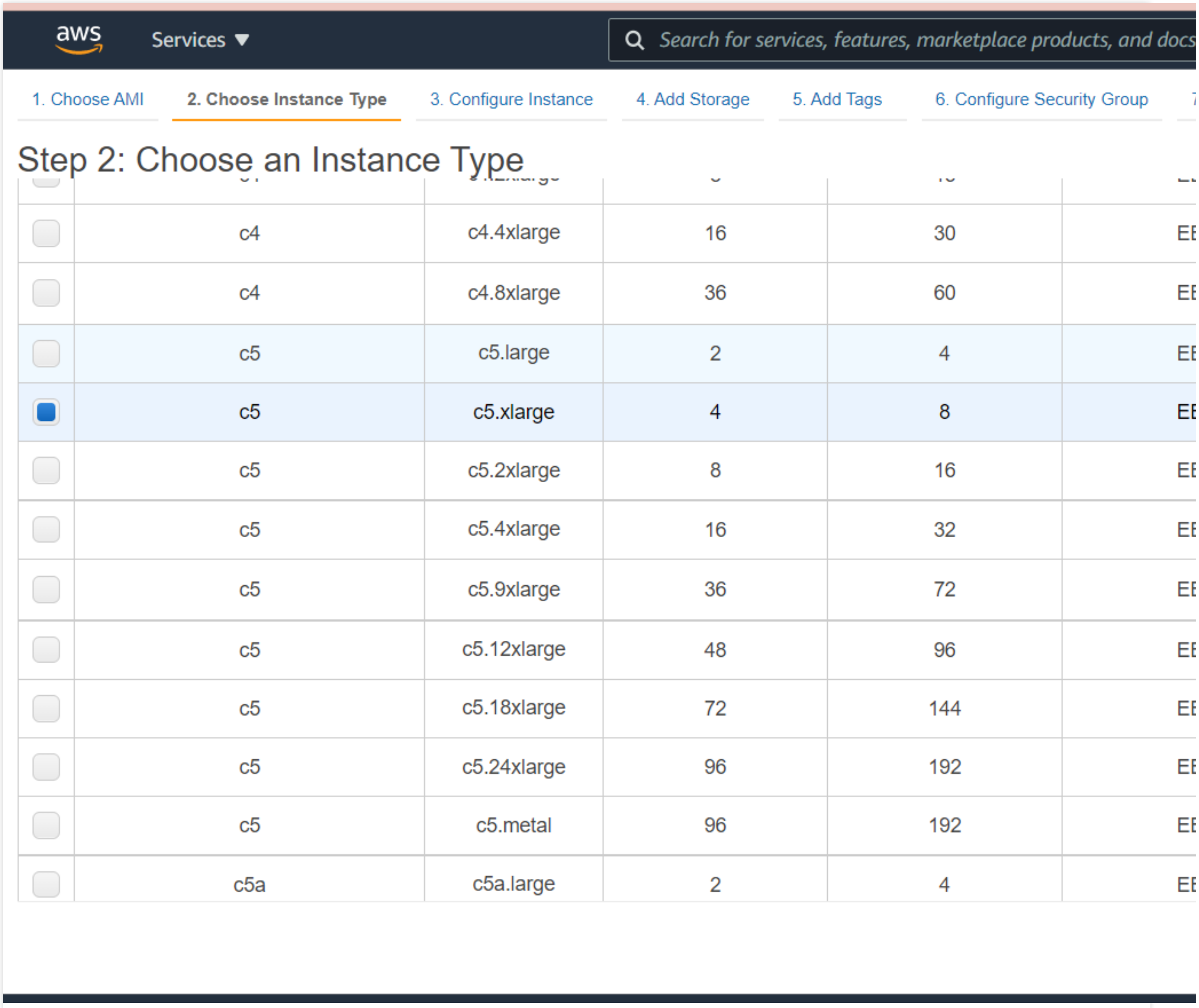

10.3 **Configure instance details**

Accept defaults

10.4 **Add Storage**

This local storage is fast but will be destroyed after you terminate instance. To start, give your instance around 3x the size of your current dataset. This provides very fast "local" storage to the instance (compared to slower S3 storage)

10.5 **Add Tags**

This is optional for better organization of instances

10.6 **Configure Security group**

Most of the time you want port 22 (ssh) to be accessible for remote connections. Jupyter Notebooks server by default runs at port 8888, so that one should also be open.

Following Justin Bois's tutorial, your security rules should look like this.

Citation: Daniel J. Pollak, Gautam Chawla, Andrey Andreev, David A. Prober Steps for setup of AWS organization, S3 data storage, and EC2 computing for using Python notebooks https://dx.doi.org/10.17504/protocols.io.rm7vz3z4xgx1/v1

This is an open access protocol distributed under the terms of the **Creative Commons Attribution License** (https://creativecommons.org/licenses/by/4.0/), which permits unrestricted use, distribution, and reproduction in any medium,

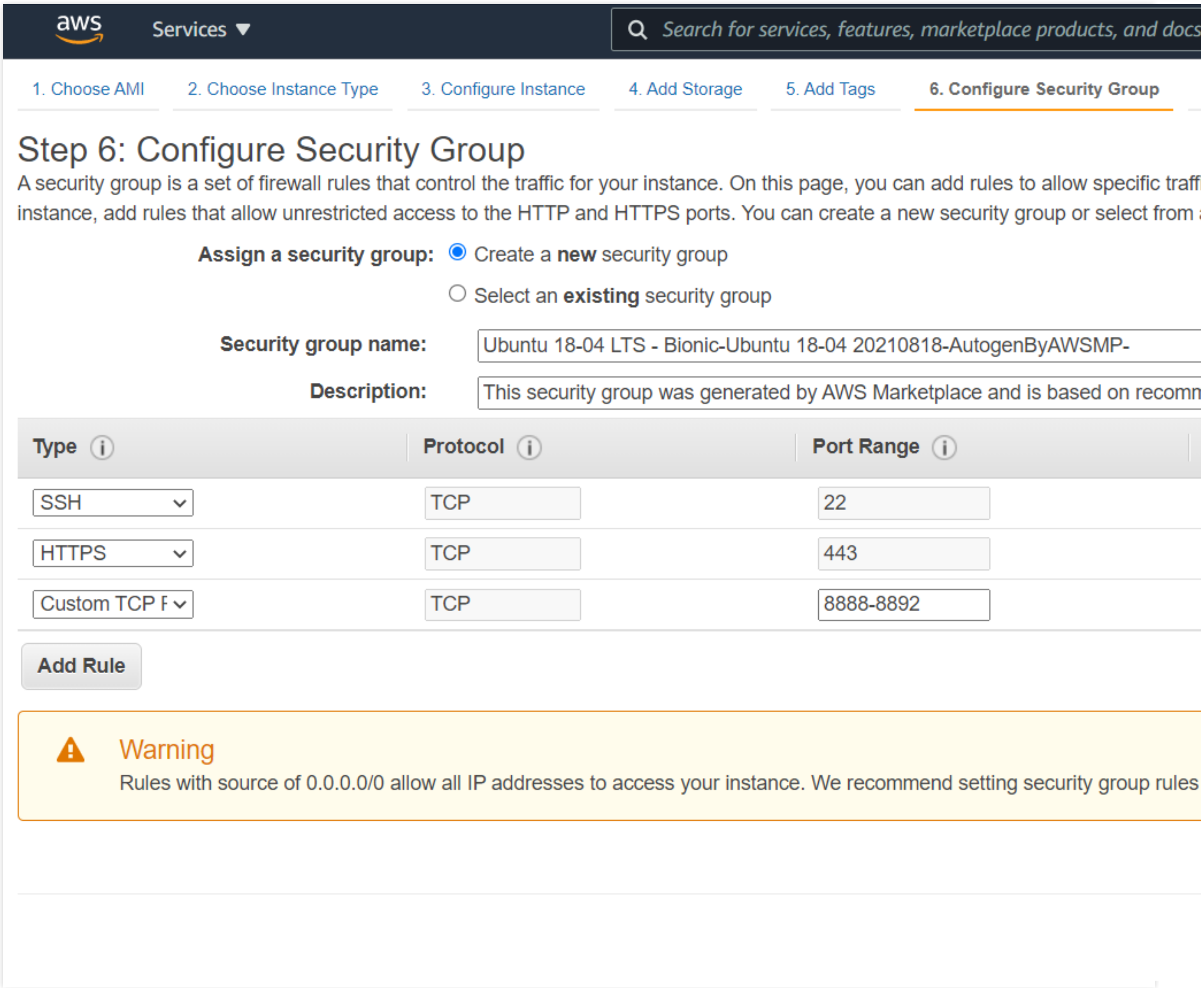

10.7 **Review Instance Launch**

Select Launch. This will open the Key Pair popup.

1. This is going to give you a “key”. Put it somewhere safe, meaning a location that is not synched to the Internet (Dropbox or Google Drive). The key will grant full access to the instance.
2. Click launch instance. It usually takes less than two minutes for instance to start.

EC2: Connecting to and using your instance

11 If you have just created the instance, it will start automatically.
If the instance has been "Stopped", start your instance. Process should take less than few minutes.

NB: at the end of the work with the instance, you can either Stop or Terminate instance.
Stopped instance will have all its memory saved in S3 storage (and you will be billed for every GB of stored data according to S3 prices). Terminated instance will have its memory completely destroyed, and you will lose any information saved in the instance.
If you don't stop the instance, you will be billed for the time it is Running, regardless of whether you are actually using it for any computations or not.
We recommend stopping instances at the end of work. Check with AWS pricing and your budget to estimate cost of storing instance data.

Citation: Daniel J. Pollak, Gautam Chawla, Andrey Andreev, David A. Prober Steps for setup of AWS organization, S3 data storage, and EC2 computing for using Python notebooks https://dx.doi.org/10.17504/protocols.io.rm7vz3z4xgx1/v1

This is an open access protocol distributed under the terms of the **Creative Commons Attribution License** (https://creativecommons.org/licenses/by/4.0/), which permits unrestricted use, distribution, and reproduction in any medium,

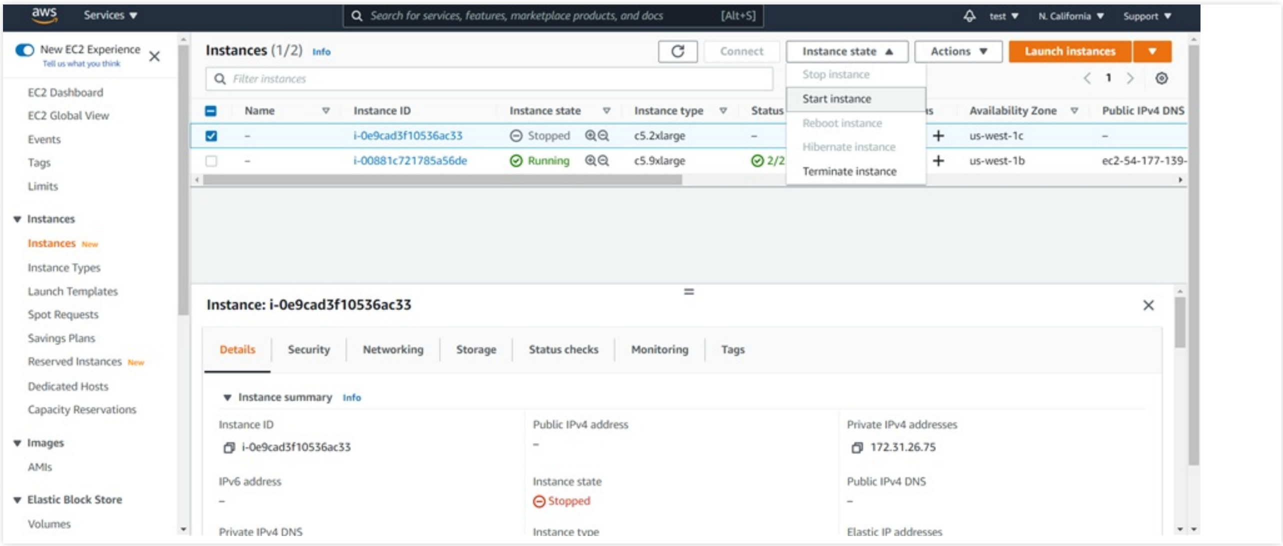

12 Click Instance ID to view the instance summary page and select connect.

13 Using web-based terminal: Select connect, which will open the terminal in another tab

Citation: Daniel J. Pollak, Gautam Chawla, Andrey Andreev, David A. Prober Steps for setup of AWS organization, S3 data storage, and EC2 computing for using Python notebooks https://dx.doi.org/10.17504/protocols.io.rm7vz3z4xgx1/v1

This is an open access protocol distributed under the terms of the **Creative Commons Attribution License** (https://creativecommons.org/licenses/by/4.0/), which permits unrestricted use, distribution, and reproduction in any medium,

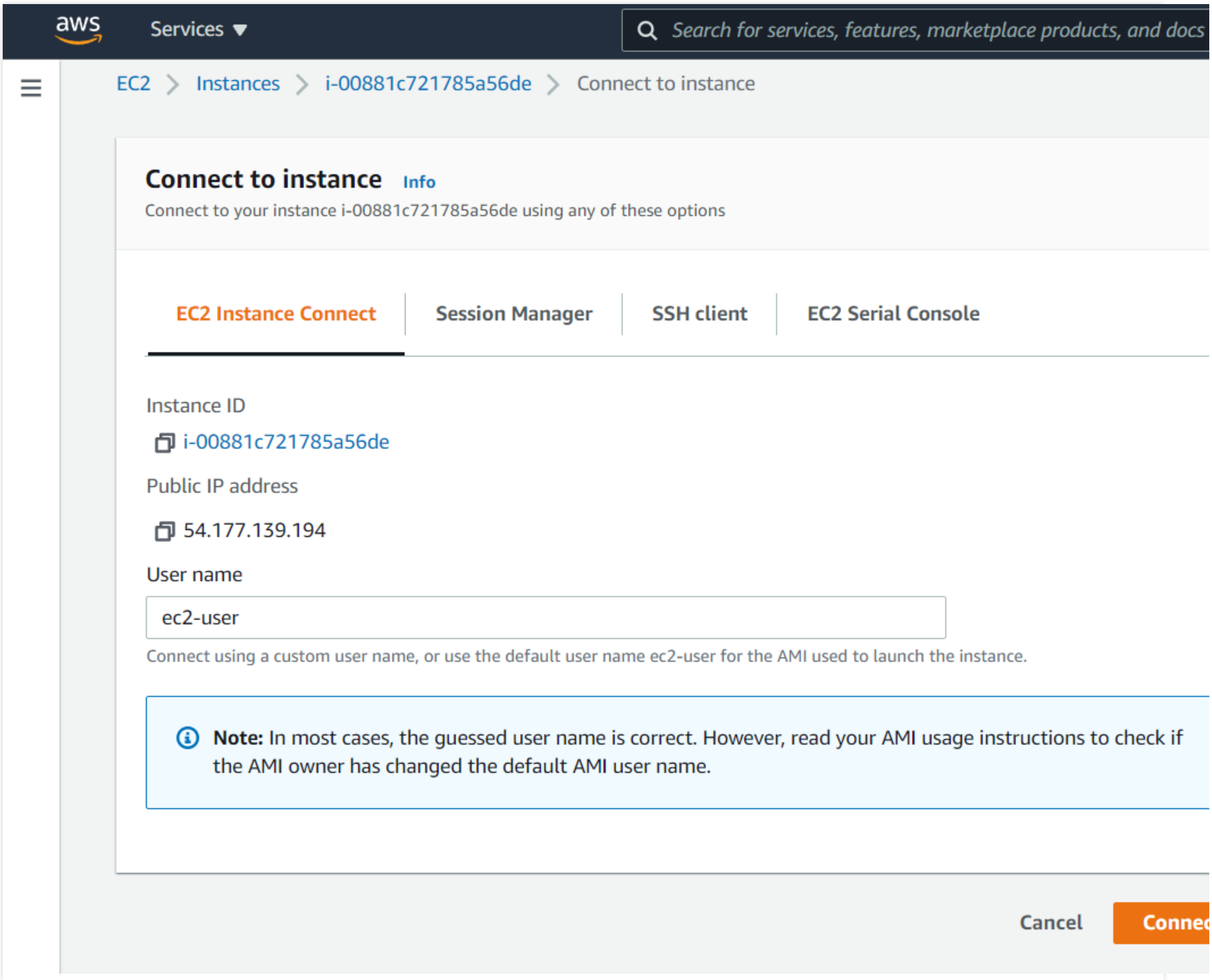

14 Using terminal / ssh:

```
chmod 600 /path/to/key.pem
ssh -i /path/to/key.pem ec2-user@[public DNS]
```

Installing dependencies

15 Instance is a virtual computer, and you should keep it organized like any other computer. For example, you might need to get code using Git or download from the internet. Cloned or downloaded git repositories go in the *git* folder, and all other downloads go in the *Downloads* folder. To create these folders use commands:

```
mkdir Downloads
mkdir git
```

16 Per Chris Albon's tutorial, install conda as follows:

Citation: Daniel J. Pollak, Gautam Chawla, Andrey Andreev, David A. Prober Steps for setup of AWS organization, S3 data storage, and EC2 computing for using Python notebooks https://dx.doi.org/10.17504/protocols.io.rm7vz3z4xgx1/v1

This is an open access protocol distributed under the terms of the **Creative Commons Attribution License** (https://creativecommons.org/licenses/by/4.0/), which permits unrestricted use, distribution, and reproduction in any medium,

16.1 On the downloads page for https://anaconda.com, right click on the download link for Linux, and select “Copy link address”.

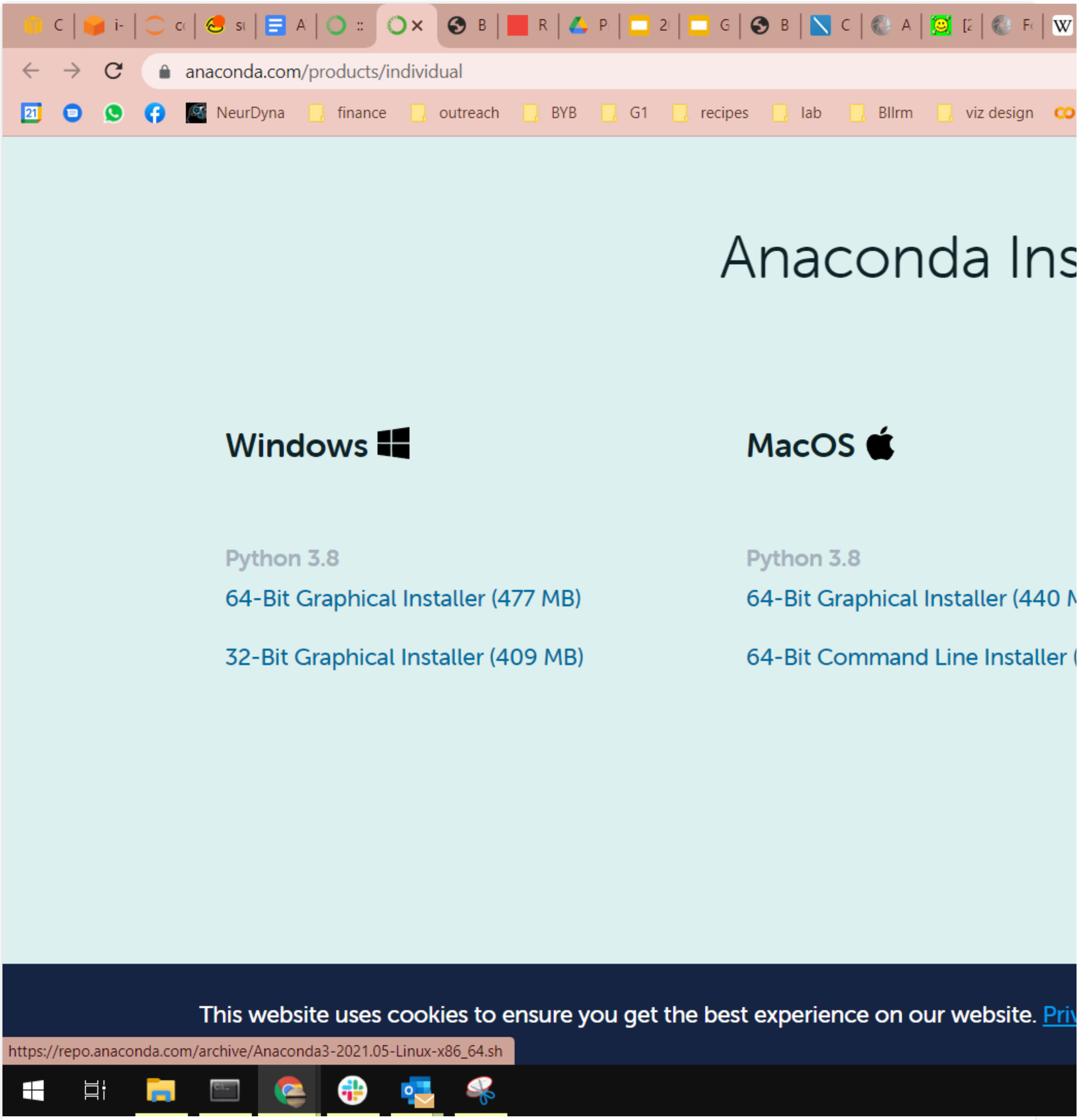

If your local machine computer runs Windows or Mac instead of Linux, you will likely be prompted with a link for your particular operating system (OS). Take care not to copy that link. **The link should end in .sh, not .exe or .pkg and look something like this:**

```
https://repo.anaconda.com/archive/Anaconda3-2021.05-Linux-x86_64.sh
```

To install use commands:

Citation: Daniel J. Pollak, Gautam Chawla, Andrey Andreev, David A. Prober Steps for setup of AWS organization, S3 data storage, and EC2 computing for using Python notebooks https://dx.doi.org/10.17504/protocols.io.rm7vz3z4xgx1/v1

This is an open access protocol distributed under the terms of the **Creative Commons Attribution License** (https://creativecommons.org/licenses/by/4.0/), which permits unrestricted use, distribution, and reproduction in any medium,

```
wget <Linux Anaconda installer link>.sh
bash <Linux Anaconda installer link>.sh
```

Follow the prompts on the screen to complete the installation

16.2 Follow the suite2p install instructions here: https://github.com/MouseLand/suite2p.
The first step is to download a YAML file, which is not necessarily clear on Linux. To download it, we will use wget again:

```
wget https://raw.githubusercontent.com/MouseLand/suite2p/main/environment.yml
```

16.3 Activate your new suite2p environment:

```
conda activate suite2p
```

16.4 Install jupyter lab:

```
conda install -c conda-forge jupyter lab
```

16.5 If you skipped Anaconda installation, use

```
pip3 install jupyter
```

and

```
 pip3 install boto3
```

16.6 Install smart_open for s3:

```
pip install smart_open[s3].
```

Make sure you install this library for s3 specifically, as specified here.

17 If your EC2 instance's OS is not Amazon Linux 2, install aws cli:

```
sudo apt install awscli
```

18 Install Julia

Citation: Daniel J. Pollak, Gautam Chawla, Andrey Andreev, David A. Prober Steps for setup of AWS organization, S3 data storage, and EC2 computing for using Python notebooks https://dx.doi.org/10.17504/protocols.io.rm7vz3z4xgx1/v1

This is an open access protocol distributed under the terms of the **Creative Commons Attribution License** (https://creativecommons.org/licenses/by/4.0/), which permits unrestricted use, distribution, and reproduction in any medium,

18.1 From your Downloads directory:

```
wget https://julialang-s3.julialang.org/bin/linux/x64/1.6/julia-1.6.2-linux-x86_64.tar.gz
```

```
tar zxvf julia-1.6.2-linux-x86_64.tar.gz
```

18.2 Install IJulia. This will allow you to run interactive julia environment inside Jupyter notebooks.
First, start Julia

```
./julia-1.6.2/bin/julia
```

And then install IJulia

```
] add IJulia
```

18.3 Within Julia interactive shell install necessary packages to work with TIFF images:

```
] import Pkg; Pkg.add("Images")
] import Pkg; Pkg.add("TiffImages")
] import Pkg; Pkg.add("FileIO")
```

Note that we are installing dependencies in a slightly different way than how we installed IJulia. These methods are equivalent.

Starting Jupyter lab

19 You are mostly finished with dependencies! You'll add more as necessary, but these are the most important. We will treat this section as though you have freshly started your EC2 instance.

20 Start conda environment:

```
conda activate suite2p
```

21 1. Open a persistent jupyter lab session:

```
nohup jupyter lab --ip 0.0.0.0 --NotebookApp.max_buffer_size=75368709120 &
```

nohup command starts the jupyter server in the background, not tied to your connection to EC2 instance via ssh. The log created by jupyter server will be written to *nohup.out* file

Citation: Daniel J. Pollak, Gautam Chawla, Andrey Andreev, David A. Prober Steps for setup of AWS organization, S3 data storage, and EC2 computing for using Python notebooks https://dx.doi.org/10.17504/protocols.io.rm7vz3z4xgx1/v1

This is an open access protocol distributed under the terms of the **Creative Commons Attribution License** (https://creativecommons.org/licenses/by/4.0/), which permits unrestricted use, distribution, and reproduction in any medium,

Several parameters are specified here so that:

1. Jupyter lab will be accessible from your browser window (--ip=0.0.0.0)
2. The memory size limit is increased (--NotebookApp.max_buffer_size=75368709120). For applications that use a lot of memory like suite2p, Kilosort, Caiman, etc Python needs to make really big arrays, and the default memory size for jupyter lab (but not for the AWS instance) is too small. For work with 20GB datasets we use ~75 GB RAM

22 Open the nohup.out file to get the token.

```
cat nohup.out
```

Copy the string following "lab?token="

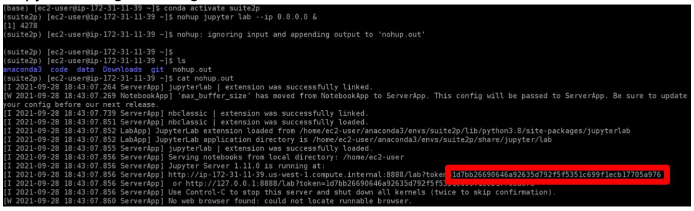

Use this token to login into the Python notebook:

Citation: Daniel J. Pollak, Gautam Chawla, Andrey Andreev, David A. Prober Steps for setup of AWS organization, S3 data storage, and EC2 computing for using Python notebooks https://dx.doi.org/10.17504/protocols.io.rm7vz3z4xgx1/v1

This is an open access protocol distributed under the terms of the **Creative Commons Attribution License** (https://creativecommons.org/licenses/by/4.0/), which permits unrestricted use, distribution, and reproduction in any medium,

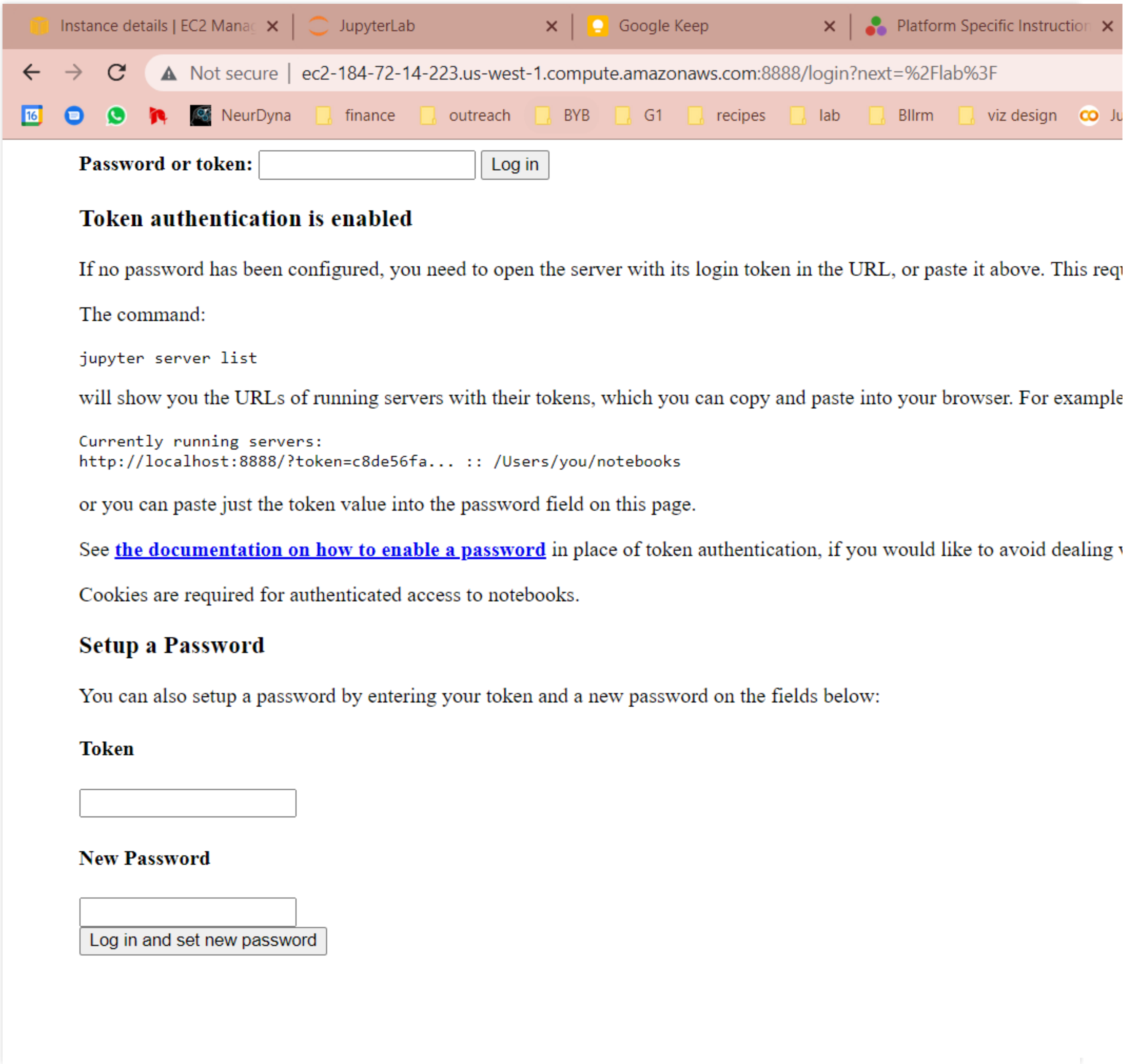

Alternatively, you might need to start up a persistent server. A persistent server will not depend on the connection between your personal computer and the EC2 instance.

22.1 The server will start a process. To find its ID, run in terminal:

1. To stop Jupyter notebook server (or lab) copy the process number and kill it:

```
ps -aux | grep python
```


Citation: Daniel J. Pollak, Gautam Chawla, Andrey Andreev, David A. Prober Steps for setup of AWS organization, S3 data storage, and EC2 computing for using Python notebooks https://dx.doi.org/10.17504/protocols.io.rm7vz3z4xgx1/v1

This is an open access protocol distributed under the terms of the **Creative Commons Attribution License** (https://creativecommons.org/licenses/by/4.0/), which permits unrestricted use, distribution, and reproduction in any medium,

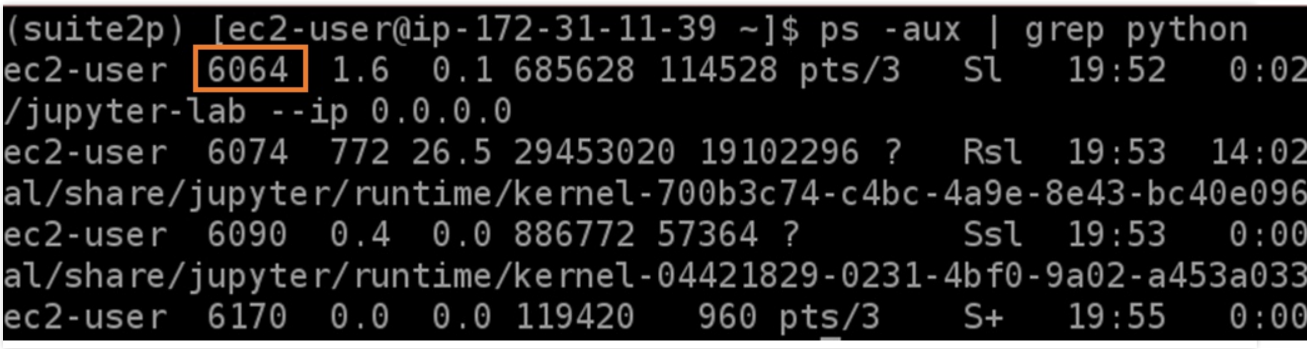

To stop Jupyter notebook server (or lab) copy the process number and kill it:

```
kill <process number>
```

S3: Uploading data *via* web-interface

23 To start, create a Bucket for your experimental data. It is important to block public access to the bucket

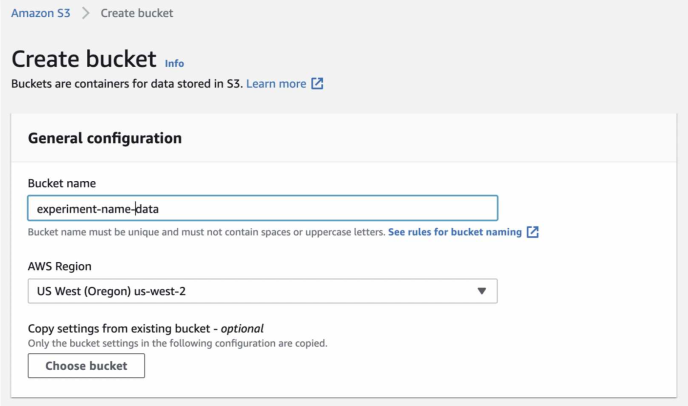

24 You can use web interface to drag-and-drop data to upload it into the bucket or into a folder within bucket

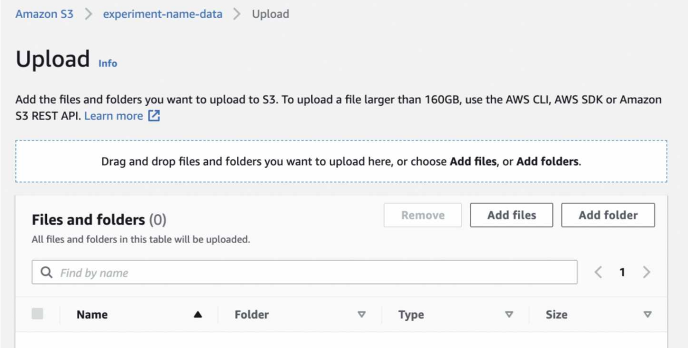

Expected speed of upload is around 10MB/s.
Largest file size allowed through web-interface is 160GB.

25 You can also use command-line interface to upload data in more automated way or upload larger files

Citation: Daniel J. Pollak, Gautam Chawla, Andrey Andreev, David A. Prober Steps for setup of AWS organization, S3 data storage, and EC2 computing for using Python notebooks https://dx.doi.org/10.17504/protocols.io.rm7vz3z4xgx1/v1

This is an open access protocol distributed under the terms of the **Creative Commons Attribution License** (https://creativecommons.org/licenses/by/4.0/), which permits unrestricted use, distribution, and reproduction in any medium,

S3: Setting up access for S3 bucket from AWS/EC2 instance

26 For each user, add their canonical ID to the bucket's access list (need to allow both list and write permissions). That will allow these users to access bucket from Python interface

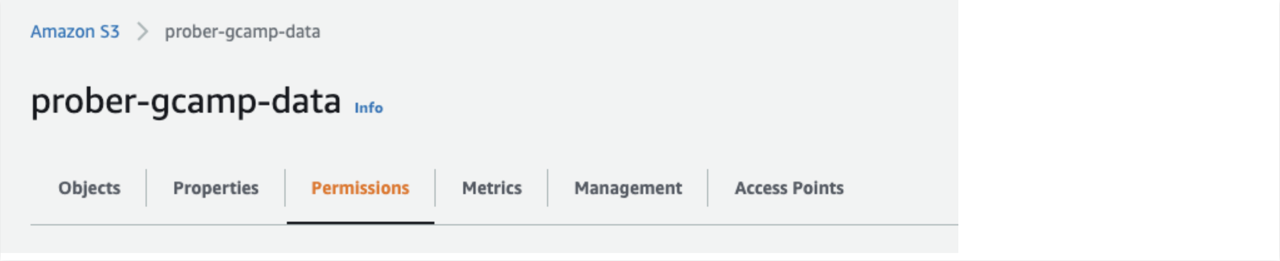

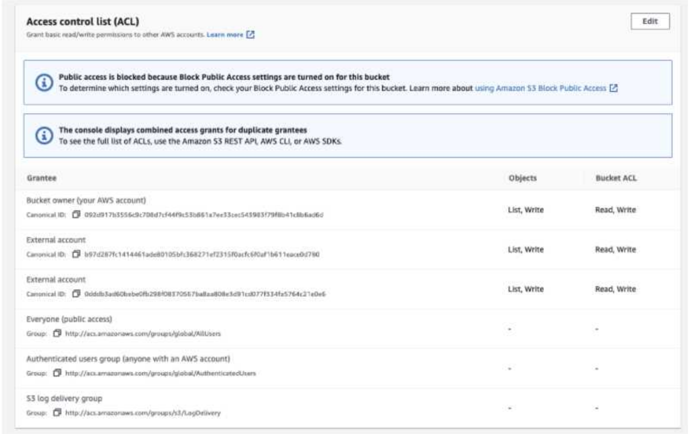

26.1 Canonical ID can be found in AWS Web Interface -> My Security Credentials -> Canonical User ID (AWS manual)

27 To allow downloading data from web browser interface, create a custom rule. It gives users who did not initially upload the data access to it through S3's web interface at https://s3.console.aws.amazon.com/

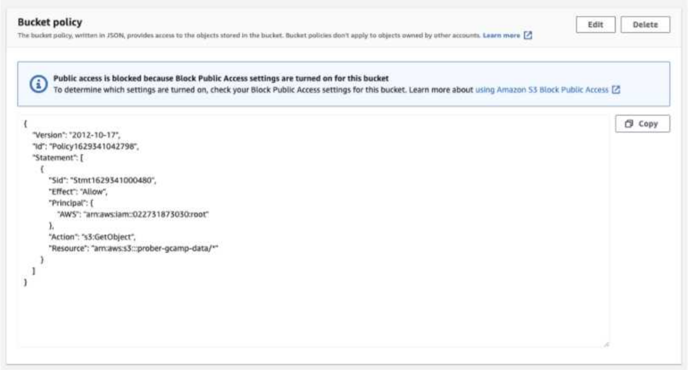

Example of policy to give user access to reading data from bucket. It has been generated using AWS Policy Generator

Here the "Principal" is the user that gets permission to read data from the bucket specified in "Resource" field

28 Each user will have to create custom key pair for your account under security Credentials → Access keys.
This will allow you to create Access Key ID and Secret Access Key.

Citation: Daniel J. Pollak, Gautam Chawla, Andrey Andreev, David A. Prober Steps for setup of AWS organization, S3 data storage, and EC2 computing for using Python notebooks https://dx.doi.org/10.17504/protocols.io.rm7vz3z4xgx1/v1

This is an open access protocol distributed under the terms of the **Creative Commons Attribution License** (https://creativecommons.org/licenses/by/4.0/), which permits unrestricted use, distribution, and reproduction in any medium,

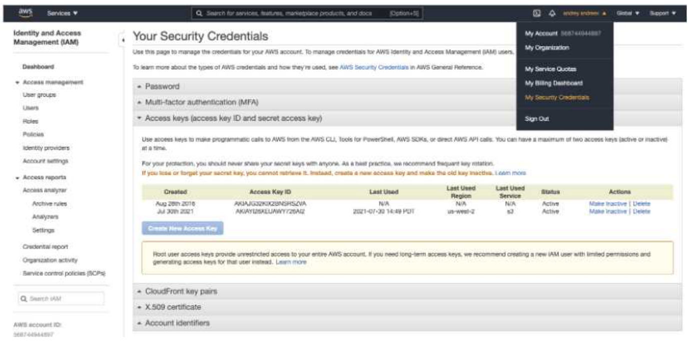

29 Connect to the EC2 instance using EC2 Connect function or *ssh*. Run *aws configure* to configure the instance. Make sure to enter the correct zone ID.

```
Access key ID: ***
Secret access key: ***
Default. region name: us-west-2
Default output format: json
```

30 While logged into the instance, run *python* to launch the Python interpreter

31 Test connection to bucket via the **boto3** library:

```
import boto3
s3 = boto3.resource('s3')
bucket = s3.Bucket('')
for obj in bucket.objects.all():
    print(obj.key)
```

This should print all items in the bucket

Accessing files from Python using boto3

32 We have just shown how to confirm that the S3 bucket is visible to your EC2 instance. To run full data processing pipelines with suite2p and other such programs, we need to go one step further, and download files into our local filesystem. Let's go through some Python code to see how it works.

[1]:

```
import os
import boto3
import matplotlib.pyplot as plt
import numpy as np
import io
import tqdm

# smart_open allows us to open large data files
from smart_open import open, register_compressor
from suite2p import run_s2p, default_ops
```


Citation: Daniel J. Pollak, Gautam Chawla, Andrey Andreev, David A. Prober Steps for setup of AWS organization, S3 data storage, and EC2 computing for using Python notebooks https://dx.doi.org/10.17504/protocols.io.rm7vz3z4xgx1/v1

This is an open access protocol distributed under the terms of the **Creative Commons Attribution License** (https://creativecommons.org/licenses/by/4.0/), which permits unrestricted use, distribution, and reproduction in any medium,

[2]:

```
# As a sanity check, list filenames from your bucket.
# We have replaced the real Bucket name with '{bucket-name}', but we have left the output in place.
s3 = boto3.resource('s3')
bucket = s3.Bucket('{bucket-name}')
for obj in bucket.objects.all():
    print(obj.key)
```

[Out]:

```
210610_920/300ms_bin1x_fullpower_1/300ms_bin1x_fullpower_1_MMStack_Pos0.ome.tif
210610_920/300ms_bin1x_fullpower_1/300ms_bin1x_fullpower_1_MMStack_Pos0_1.ome.tif
210610_920/300ms_bin1x_fullpower_1/300ms_bin1x_fullpower_1_MMStack_Pos0_2.ome.tif
210610_920/300ms_bin1x_fullpower_1/300ms_bin1x_fullpower_1_MMStack_Pos0_3.ome.tif
```

[3]:

```
# Next, we are going to download our files into the local filesystem. This will be very fast because we chose an
instance type with fast internet.
# Make data directory
data_dir = "../data/210610_920/300ms_bin1x_fullpower_1/"
ifnot os.path.isdir(data_dir):
    os.makedirs(data_dir)
```

[4]:

```
# Write all datafiles locally
# ImageJ acquires large data by splitting it into 4GB files

for fname in tqdm.tqdm(["300ms_bin1x_fullpower_1_MMStack_Pos0.ome.tif",
"300ms_bin1x_fullpower_1_MMStack_Pos0_1.ome.tif",
"300ms_bin1x_fullpower_1_MMStack_Pos0_2.ome.tif",
"300ms_bin1x_fullpower_1_MMStack_Pos0_3.ome.tif"]):


# Combine path and name
    data_file = os.path.join(data_dir, fname)

# If not a file, write it. Expected speed is 10GBps between EC2 and S3, so 4GB file should be downloaded in 3
sec
ifnot os.path.isfile(data_file):
# Get object from S3
        s3.meta.client.download_file(
'prober-gcamp-data',
            os.path.join("210610_920/300ms_bin1x_fullpower_1/", fname),
            data_file
        )

        print("wrote file successfully")
else:
        print("file already exists")
```

[5]:

```
# Populates ops with the default options, except for batch_size, which we will make smaller to ensure the kernel
does not shut down.
ops = default_ops()
ops['batch_size'] = 100
# Only run on specified tiffs
db = {
'h5py': [], # a single h5 file path
'h5py_key': 'data',
'look_one_level_down': False, # Whether to look in ALL subfolders when searching for tiffs
```


Citation: Daniel J. Pollak, Gautam Chawla, Andrey Andreev, David A. Prober Steps for setup of AWS organization, S3 data storage, and EC2 computing for using Python notebooks https://dx.doi.org/10.17504/protocols.io.rm7vz3z4xgx1/v1

This is an open access protocol distributed under the terms of the **Creative Commons Attribution License** (https://creativecommons.org/licenses/by/4.0/), which permits unrestricted use, distribution, and reproduction in any medium,

```
'data_path': [data_dir],
'tiff_list': [elem for elem in os.listdir(data_dir) if elem.endswith("tif")]
}

# Run suite2p
opsEnd = run_s2p(ops=ops, db=db)
```

[Out]:

```
{'h5py': [], 'h5py_key': 'data', 'look_one_level_down': False, 'data_path':
['../data/210610_920/300ms_bin1x_fullpower_1/'], 'tiff_list': ['300ms_bin1x_fullpower_1_MMStack_Pos0.ome.tif',
'300ms_bin1x_fullpower_1_MMStack_Pos0_1.ome.tif', '300ms_bin1x_fullpower_1_MMStack_Pos0_2.ome.tif',
'300ms_bin1x_fullpower_1_MMStack_Pos0_3.ome.tif']}
FOUND BINARIES AND OPS IN ['../data/210610_920/300ms_bin1x_fullpower_1/suite2p/plane0/ops.npy']
>>>>>>>>>>>>>>>>>>>>> PLANE 0 <<<<<<<<<<<<<<<<<<<<<<
NOTE: not registered / registration forced with ops['do_registration']>1
      (no previous offsets to delete)
----------- REGISTRATION

registering 2000 frames
Reference frame, 103.47 sec.
Registered 400/2000 in 188.83s
Registered 800/2000 in 376.80s
Registered 1200/2000 in 564.32s
Registered 1600/2000 in 751.31s
Registered 2000/2000 in 939.14s

/home/ec2-user/anaconda3/envs/suite2p/lib/python3.8/site-packages/suite2p/registration/register.py:44:
RuntimeWarning: invalid value encountered in true_divide
  dxy = dxy / dxy.mean()

added enhanced mean image
----------- Total 1269.39 sec
```

Creating AMI (Amazon Machine Image) from configured instance

33 It is useful to save your configured machine image for later. Creating Image will save all packages installed, all data, and all private/sensitive information too.

33.1 Go to Actions → Image and Templates → Create Image

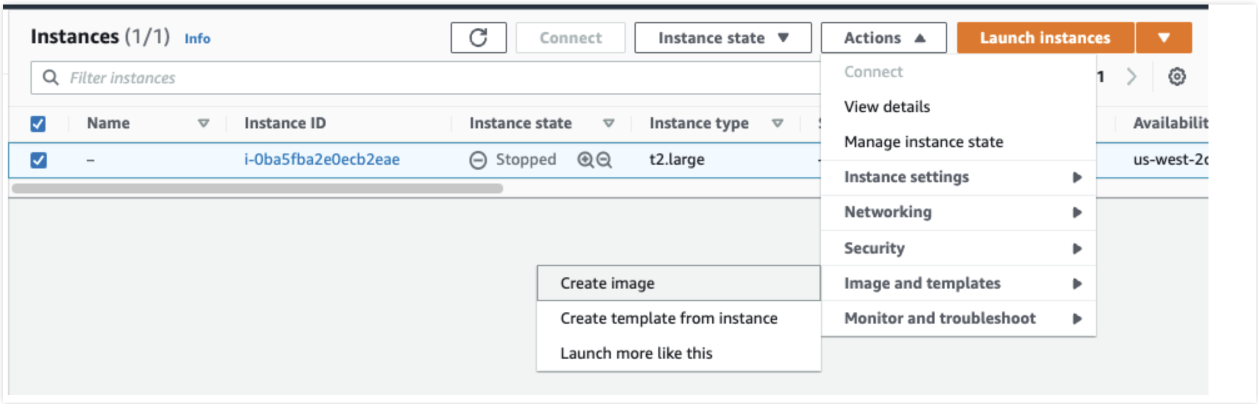

33.2
1. Pick an appropriate name (for example “conda-image” for version with full installation of Python and Conda) and click Create Image
2. NB: the image will be created with all the data saved on the “local” disk. So if you have instance with 100GB of data saved, data will be added to the image as well

Citation: Daniel J. Pollak, Gautam Chawla, Andrey Andreev, David A. Prober Steps for setup of AWS organization, S3 data storage, and EC2 computing for using Python notebooks https://dx.doi.org/10.17504/protocols.io.rm7vz3z4xgx1/v1

This is an open access protocol distributed under the terms of the **Creative Commons Attribution License** (https://creativecommons.org/licenses/by/4.0/), which permits unrestricted use, distribution, and reproduction in any medium,

Note that images are stored on S3 storage service, and you will be charged for storing every image

Now you can launch an identical instance of your customized AMI by selecting your image from “My AMIs” when spinning up a new EC2 instance.

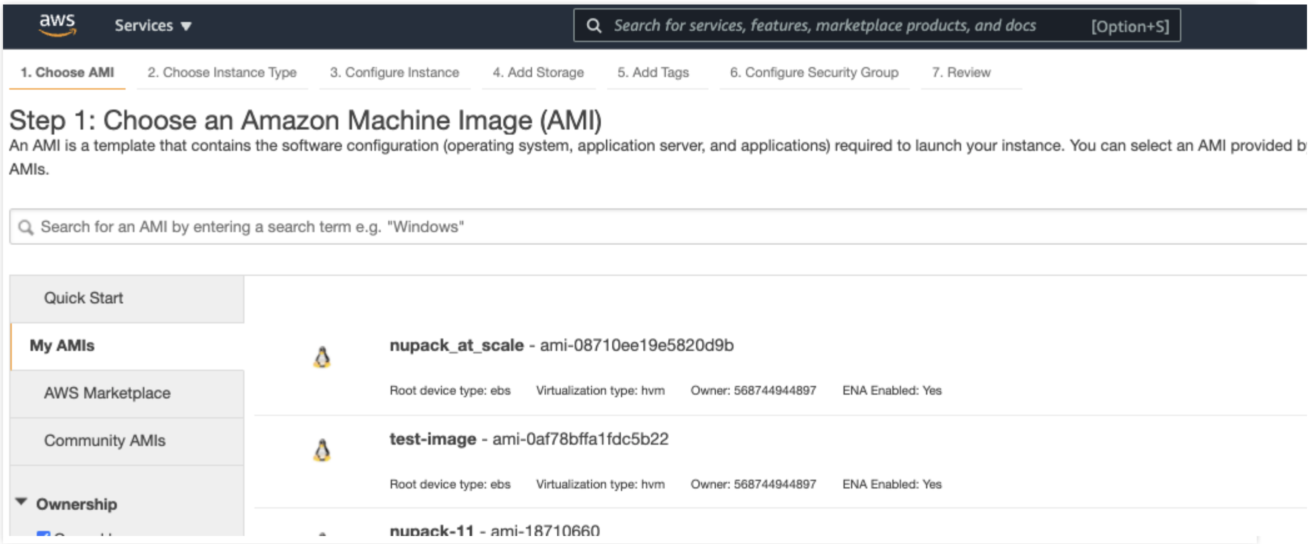

Citation: Daniel J. Pollak, Gautam Chawla, Andrey Andreev, David A. Prober Steps for setup of AWS organization, S3 data storage, and EC2 computing for using Python notebooks https://dx.doi.org/10.17504/protocols.io.rm7vz3z4xgx1/v1

This is an open access protocol distributed under the terms of the **Creative Commons Attribution License** (https://creativecommons.org/licenses/by/4.0/), which permits unrestricted use, distribution, and reproduction in any medium,
