# Supplementary material for: First steps into the cloud: Using Amazon data storage and computing with Python notebooks
Source: PLoS One. 2023 Feb 9;18(2):e0278316. doi: 10.1371/journal.pone.0278316 (PMC9910747; doi:10.1371/journal.pone.0278316)
Supplement: S1 Data — (DOCX) [file pone.0278316.s002.docx]

Supporting code and calcium imaging data: doi.org/10.22002/6ejqf-qm267.
