# Supplementary material for: First steps into the cloud: Using Amazon data storage and computing with Python notebooks
Source: PLoS One. 2023 Feb 9;18(2):e0278316. doi: 10.1371/journal.pone.0278316 (PMC9910747; doi:10.1371/journal.pone.0278316)
Supplement: Attachment — Submitted filename: 220216_andreev_response.docx [file pone.0278316.s003.docx]

Dear PLOS One editor,

We are thankful for the opportunity to present our work and communicate with larger research community about practical applications and challenges of cloud computing on commercial platforms such as Amazon Web Services.

As suggested by the editor and the reviewer, we have performed major revision of the paper to provide more details and insights for the readers even not familiar with cloud computing. Specifically, we expanded discussion of every section: from concerns about budgeting and billing, to description of process to start virtual machines.

Below we provide details response to reviewer’s comments.

Additional Editor Comments:
The manuscript too brief to be qualified as a journal paper but I like the topic. Cloud computing has practical importance. The author should do a major revision of the paper. The paper should present sufficient information so that a reader can reproduce the work, and also provide sufficient insights so that a reader can appreciate the problems and contributions.

We have expanded text to include more details in every section. Cloud infrastructure is discussed in more details to help those new to the concept of distributed computing, and specifically to Amazon’s cloud architecture. We have expanded section about organization of cloud computing for the whole lab, discussing how users can have separate budgets and access rights. We focused on how researchers can avoid accidentally wasting money. We have also included a step-by-step guide on setting up computing environment, so that just reading the paper (even before opening the protocol.io document) users can get valuable insights and reference material. We have also included more screenshots. We have discussed how python language and Jupyter notebooks can be leveraged to increase reproducibility of the research.

6. Review Comments to the Author

Although it is understood that such articles are meant to be brief, the current article is too brief. […] But the main article should have substantial amount of information and description to allow users to follow through and understand each main step of analysis with reference to the validated case (of zebrafish data). In the article’s current state, a person into his/her “first steps” will not understand well. Please improve on this.

We have expanded the amount of text covering motivation and explanation for each section in the manuscript. We provided more detailed discussion of concepts used in cloud computing and Amazon cloud, specifically.

In this article and/or the supporting file, it will be good to also include some potential issues that the user might face while following through the steps, as well as troubleshooting methods.

We have highlighted several issues that users can face: inability to connect to new instance (that can be fixed by firewall settings – we provide information) and budget over-runs (which can be alleviated by managing budgets and users – we provide discussion of some tools for that)

As this is a protocol for “First steps into the cloud” as described by the title, the author should address the concerns that a beginner might have regarding cloud computing. For example, although the protocol is focused on Amazon, you should also compare AWS to other IaaS providers. […]

We have briefly compared Amazon with other cloud providers; however, we note that all providers are different from each other, and translating knowledge between them is not easy. Hence, here we explicitly focus on Amazon provider as they provide the largest suite of tools, and we have the most experience with Amazon.

3rd paragraph of Introduction: “Replicability challenges faced by academics are formidable”. The word “formidable” is unclear and confusing. Is it in a good way, or bad way?

3rd paragraph of sub-heading Introduction: “As the biological sciences become more computationally- and data-driven,collaborators and information consumers increasingly try to replicate both hardware and software”. The word “replicate” is not a good choice of word here. Replicate suggests the need to make exact copies of hardware and software, which may not be necessary. Please rephrase.

Edited language

1st paragraph of sub-heading Materials and methods: “AWS uses a framework called Organizations to control who can use AWS resources outside of the Organization administrator”. Please use academic writing. It sounds very much like the author is casually speaking.

Similarly, in the same paragraph, the author wrote “Which AWS services will we be using?” before answering the question. Having ideas/points written in the form of questions is not appropriate for a proper article. Please change it.

In fact, there are other instances where the English expressions used are too casual. Please check through the entire article and change to academic writing style.

Significantly changed language to be more within academic style, and more precise

Last bullet-point of sub-heading Costs and use cases: “For a primer on best practices for data storage, see [1].” Please include the authors’ names every time you include a reference. This is not only for better academic writing, but also allows readers to know the work immediately without going around to find that reference.

Changed references to PLOS style

For sub-heading Costs and use cases: Costs and use cases seem to be very different topics. Unless the author is trying to relate costs to use cases (which I do not observe), it should be kept as separate sub-headings.

Edited to split costs and applications in separate sections
