# Supplementary material for: First steps into the cloud: Using Amazon data storage and computing with Python notebooks
Source: PLoS One. 2023 Feb 9;18(2):e0278316. doi: 10.1371/journal.pone.0278316 (PMC9910747; doi:10.1371/journal.pone.0278316)
Supplement: Attachment — Submitted filename: 20221103_rebuttal.docx [file pone.0278316.s004.docx]

To the editor,

We thank the editor and the reviewers for feedback and timely response to our re-submission.

We have amended the manuscript by adding DOI links to the data (hosted on CaltechDATA archive) and Protocols.IO.

We would like to specifically address comment from the Reviewer #2:

The screenshots you presented in this paper is not clear and create some confusion. Try to avoid use of screenshots.

We feel that using screenshots in the main article text helps us to “demystify” cloud computing interfaces, which is the main goal of the article. We reviewed the captions to make sure they are as clear as possible and contain enough information to guide the reader.
